# 3,3′-({4-[(4,5-Di­cyano-1*H*-imidazol-2-yl)diazen­yl]phen­yl}imino)­dipropionic acid

**DOI:** 10.1107/S1600536813011185

**Published:** 2013-04-27

**Authors:** Roberto Centore, Vincenzo Piccialli, Angela Tuzi

**Affiliations:** a Dipartimento di Scienze Chimiche, Università degli Studi di Napoli ’Federico II’, Complesso di Monte S. Angelo, Via Cinthia, 80126 Napoli, Italy

## Abstract

The title compound, C_17_H_15_N_7_O_4_, is a push–pull non-linear optical chromophore containing a di­alkyl­amino donor group and the di­cyano­imidazolyl acceptor separated by a π-conjugated path. The benzene and imidazole rings are not coplanar, making a dihedral angle of 10.0 (2)°. In the crystal, mol­ecules are linked by an extended set of hydrogen bonds and several motifs are recognized. Pairs of mol­ecules are held together by hydrogen bonding between carb­oxy O—H donor groups and diazenyl N-atom acceptors, forming *R*_2_^2^(24) ring patterns across inversion centres. Four-mol­ecule *R*_4_^4^(28) ring motifs are formed, again across inversion centres, through hydrogen bonding involving carb­oxy O—H donor groups and diazenyl and imidazole N-atom acceptors. Four-mol­ecule *R*_4_^4^(42) patterns are formed among mol­ecules related by translation and involve carb­oxy O—H and imidazole N—H donor groups with carbonyl O-atom and imidazole N-atom acceptors.

## Related literature

For a general survey of advanced materials based on heterocycles, see: Dalton (2002 ▶); Heeger (2010 ▶). For semiconductor, optoelectronic and piezoelectric materials containing heterocycles, see: Centore, Ricciotti *et al.* (2012 ▶); Centore, Concilio *et al.* (2012 ▶). For structural analysis of conjugation in heterocycle-based organic mol­ecules, see: Carella, Centore, Fort *et al.* (2004 ▶); Gainsford *et al.* (2008 ▶). For structural and theoretical analysis of conjugation in metallorganic compounds containing heterocycles, see: Takjoo *et al.* (2011 ▶); Takjoo & Centore (2013 ▶). For theoretical computations on π-conjugated compounds, see: Capobianco *et al.* (2012 ▶, 2013 ▶). For the synthesis of related heterocyclic compounds, see: Carella, Centore, Sirigu *et al.* (2004 ▶); Piccialli *et al.* (2013 ▶); Centore, Fusco, Capobianco *et al.* (2013 ▶). For the local packing modes of non-linear optical chromophores see: Thallapally *et al.* (2002 ▶); Centore & Piccialli (2012 ▶); Centore, Piccialli & Tuzi (2013 ▶). For hydrogen bonding in crystal structures, see: Allen *et al.* (1999 ▶); Steiner (2002 ▶); Centore, Jazbinsek *et al.* (2012 ▶); Centore, Fusco, Jazbinsek *et al.* (2013 ▶). For the synthesis of similar diazo-chromophores, see: Centore *et al.* (2007 ▶).
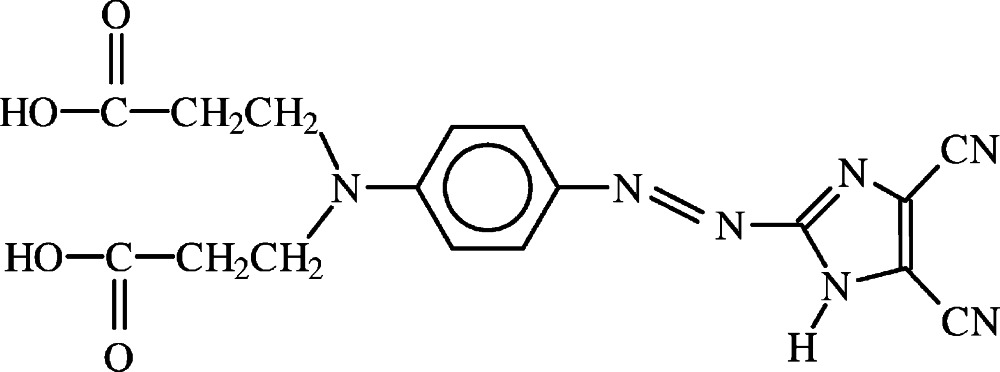


## Experimental

### 

#### Crystal data

C_17_H_15_N_7_O_4_
*M* *_r_* = 381.36
Triclinic, 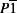

*a* = 6.895 (5) Å
*b* = 10.443 (3) Å
*c* = 13.373 (3) Å
α = 105.40 (2)°
β = 103.96 (4)°
γ = 104.84 (4)°
*V* = 846.5 (7) Å^3^
*Z* = 2
Mo *K*α radiation
μ = 0.11 mm^−1^
*T* = 293 K
0.26 × 0.13 × 0.07 mm

#### Data collection

Enraf–Nonius MACH3 diffractometer
4256 measured reflections
4082 independent reflections
1220 reflections with *I* > 2σ(*I*)
*R*_int_ = 0.074
1 standard reflections every 120 min intensity decay: none

#### Refinement

*R*[*F* ^2^ > 2σ(*F* ^2^)] = 0.075
*wR*(*F* ^2^) = 0.216
*S* = 0.91
4082 reflections
262 parameters
H atoms treated by a mixture of independent and constrained refinement
Δρ_max_ = 0.30 e Å^−3^
Δρ_min_ = −0.33 e Å^−3^

### 

Data collection: *MACH3/PC* Software (Nonius, 1996 ▶); cell refinement: *CELLFITW* (Centore, 2004 ▶); data reduction: *XCAD4* (Harms & Wocadlo, 1995 ▶); program(s) used to solve structure: *SIR97* (Altomare *et al.*, 1999 ▶); program(s) used to refine structure: *SHELXL97* (Sheldrick, 2008 ▶); molecular graphics: *ORTEP-3 for Windows* (Farrugia, 2012 ▶) and *Mercury* (Macrae *et al.*, 2006 ▶); software used to prepare material for publication: *WinGX* (Farrugia, 2012 ▶).

## Supplementary Material

Crystal structure: contains datablock(s) global, I. DOI: 10.1107/S1600536813011185/bx2439sup1.cif

Structure factors: contains datablock(s) I. DOI: 10.1107/S1600536813011185/bx2439Isup2.hkl

Supplementary material file. DOI: 10.1107/S1600536813011185/bx2439Isup3.cml

Additional supplementary materials:  crystallographic information; 3D view; checkCIF report

## Figures and Tables

**Table 1:** Hydrogen-bond geometry (Å, °)

*D*—H⋯*A*	*D*—H	H⋯*A*	*D*⋯*A*	*D*—H⋯*A*
O2—H2⋯N5^i^	0.80 (6)	2.12 (6)	2.896 (6)	162 (6)
O3—H3⋯N3^ii^	0.88 (5)	1.89 (6)	2.750 (6)	165 (5)
N4—H4⋯O4^iii^	0.81 (5)	1.98 (5)	2.740 (6)	156 (5)

Symmetry codes: (i) 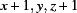; (ii) 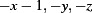; (iii) 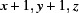.

**Fig. 1.**
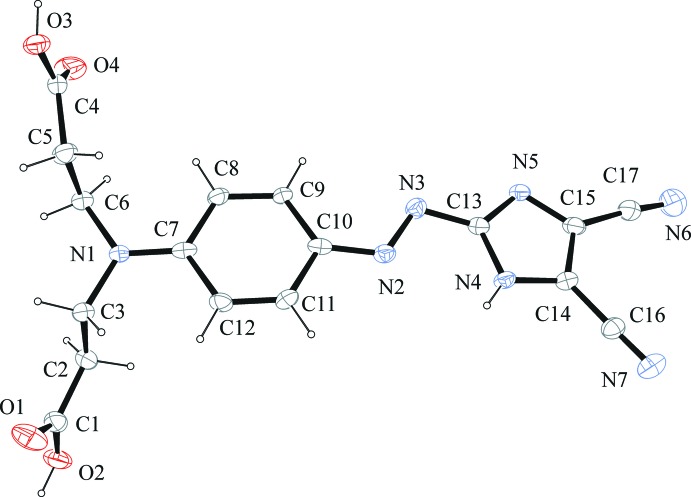
ORTEP view of the molecular structure of the title compound. Thermal ellipsoids are drawn at 30% probability level.

**Fig. 2.**
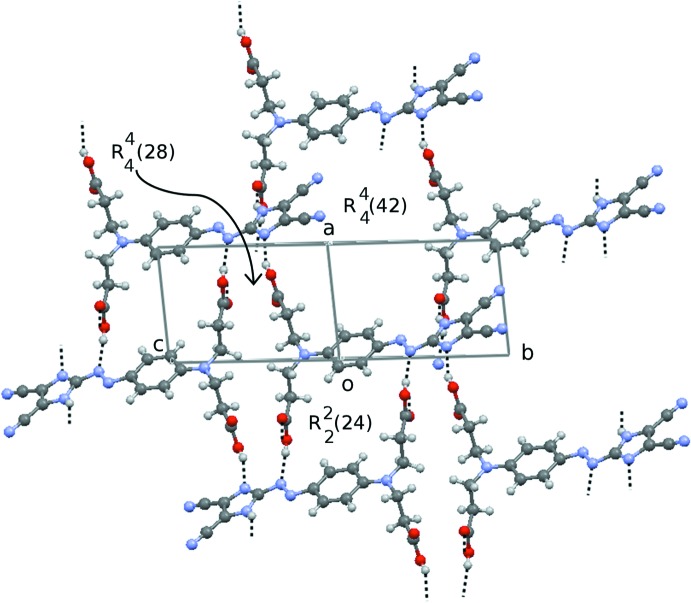
Packing of the title compound viewed along b + c. H bonds are represented by dashed lines.

## supplementary crystallographic information

### Comment

Aromatic heterocycles are playing a fundamental role in modern material chemistry as building blocks of conjugated active molecules in many fields of organic electronics and optoelectronics: conjugated conducting polymers and organic solar cells (Heeger, 2010), organic field-effect transistors (Centore, Ricciotti *et al.*, 2012), nonlinear optically active and piezoelectric compounds (Dalton, 2002; Centore, Concilio *et al.*, 2012). In those fields, the chemical investigation is devoted to the synthesis of new molecules or conjugated polymers containing heterocyclic moieties and to the measurement of their spectroscopic and electronic properties relevant for device performances. However, also the structural investigation of the molecules is a relevant point for the evaluation of the structural parameters related to the conjugation (Carella, Centore, Fort *et al.*, 2004; Gainsford *et al.*, 2008; Capobianco *et al.*, 2012; Capobianco *et al.*, 2013). The rationalization of the local packing modes of chromophore units (Thallapally *et al.*, 2002; Centore & Piccialli, 2012; Centore, Piccialli & Tuzi, 2013) is another crucial point, because many properties required for optimum device performances (*e. g.* electron mobility) critically depend on the packing not less than on strictly molecular properties. In our research group we are interested in the synthesis of new heterocyclic compounds, including metal containing heterocyclic compounds (Takjoo *et al.*, 2011; Takjoo & Centore, 2013), for applications as advanced materials and bioactive compounds (Piccialli *et al.*, 2013), and in the analysis of crystal structures controlled by the formation of H bonds (Centore, Jazbinsek *et al.*, 2012; Centore, Fusco, Jazbinsek *et al.*, 2013). Following these issues, we report, in the present paper, the structural investigation of the title compound, shown in the Scheme. The title compound is a typical push-pull azo-dye, containing the dialkylamino as donor group and two cyano acceptor groups. Moreover, the cyano groups are attached to an electron poor imidazole ring. The chromophore unit has been used in the synthesis of polymers showing quadratic NLO behaviour (Carella, Centore, Sirigu *et al.*, 2004).

The molecular structure is shown in Fig. 1. The geometry around the donor N1 atom is substantially planar indicating *sp*^2^ hybridization (the sum of valence angles at N1 is 360°) and the pattern of bond lenghts within the adjacent phenyl ring shows a certain degree of quinoidal character. All these structural features are in accordance with the expected π conjugation and push-pull character of the chromophore group.

The two aromatic rings are not coplanar, the dihedral angle between the mean planes being 10.0 (2)°; the π-conjugated part of the molecule has a slighlty curved shape, as the result of small torsions around the bonds C10—N2, N2–N3 and N3–C13.

The molecules of the title compound have several H bonding donor and acceptor groups, and the crystal packing is dominated by the formation of H bonds, Table 1. Several H bonding motifs are recognized in the crystal packing (Allen *et al.*, 1999; Steiner, 2002) and some of them are shown in Fig. 2. Couples of molecules are held by H bonding between carboxy O–H donors and azo N acceptors, forming *R*^2^_2_(24) ring patterns across inversion centres. Four-molecule ring motifs *R*^4^_4_(28) are formed, again across inversion centres, through H bonding involving carboxy O–H donors and azo and imidazole N acceptors. Four-molecule ring motifs *R*^4^_4_(42) are formed, among molecules related by translation, through H bonding involving carboxy O–H and imidazole N–H donors and carbonyl and imidazole N acceptors.

### Experimental

The title compound was prepared by diazotization of 2-amino-4,5-dicyanoimidazole followed by coupling with *N,N*-(*bis*2-carboxyethylamino)aniline. The procedure of diazo-coupling is analogous to that we have already described for the synthesis of similar diazo-chromophores (Centore *et al.*, 2007). Purification was obtained by recrystallization from hot acetic acid. The final yield for the diazotization/coupling step was 91%. Mp. 230 °C (dec). Single crystals were obtained by slow evaporation from acetic acid solutions. ^1^H-NMR (DMSO-d_6_) δ 2.45 (tr, 4H), 3.61 (tr, 4H), 6.79 (d, 2H, J = 9 Hz), 7.69 (d, 2H, J = 9 Hz). λ_max_(DMF) = 452 nm, ε_max_(DMF)= 2.5 × 10 ^4^ L mol^-1^cm^-1^.

### Refinement

The H atoms of the carboxy groups and of the imidazole ring were located in difmaps and their coordinates were refined. All other H atoms were generated stereochemically and were refined by the riding model. For all H atoms *U*_iso_=1.2×*U*_eq_ of the carrier atom was assumed.

### Crystal data

**Table:**

C_17_H_15_N_7_O_4_	*Z* = 2
*M**_r_* = 381.36	*F*(000) = 396
Triclinic, *P*1	*D*_x_ = 1.496 Mg m^−^^3^
*a* = 6.895 (5) Å	Mo *K*α radiation, λ = 0.71073 Å
*b* = 10.443 (3) Å	Cell parameters from 25 reflections
*c* = 13.373 (3) Å	θ = 7.2–9.8°
α = 105.40 (2)°	µ = 0.11 mm^−^^1^
β = 103.96 (4)°	*T* = 293 K
γ = 104.84 (4)°	Plate, red
*V* = 846.5 (7) Å^3^	0.26 × 0.13 × 0.07 mm

### Data collection

**Table:**

Enraf–Nonius MACH3 diffractometer	*R*_int_ = 0.074
Radiation source: fine-focus sealed tube	θ_max_ = 28.0°, θ_min_ = 1.7°
Graphite monochromator	*h* = −9→8
Nonprofiled ω scans	*k* = −13→13
4256 measured reflections	*l* = 0→17
4082 independent reflections	1 standard reflections every 120 min
1220 reflections with *I* > 2σ(*I*)	intensity decay: none

### Refinement

**Table:**

Refinement on *F*^2^	Primary atom site location: structure-invariant direct methods
Least-squares matrix: full	Secondary atom site location: difference Fourier map
*R*[*F*^2^ > 2σ(*F*^2^)] = 0.075	Hydrogen site location: inferred from neighbouring sites
*wR*(*F*^2^) = 0.216	H atoms treated by a mixture of independent and constrained refinement
*S* = 0.91	*w* = 1/[σ^2^(*F*_o_^2^) + (0.072*P*)^2^] where *P* = (*F*_o_^2^ + 2*F*_c_^2^)/3
4082 reflections	(Δ/σ)_max_ < 0.001
262 parameters	Δρ_max_ = 0.30 e Å^−^^3^
0 restraints	Δρ_min_ = −0.33 e Å^−^^3^

### Special details

**Table:**

Geometry. All e.s.d.'s (except the e.s.d. in the dihedral angle between two l.s. planes) are estimated using the full covariance matrix. The cell e.s.d.'s are taken into account individually in the estimation of e.s.d.'s in distances, angles and torsion angles; correlations between e.s.d.'s in cell parameters are only used when they are defined by crystal symmetry. An approximate (isotropic) treatment of cell e.s.d.'s is used for estimating e.s.d.'s involving l.s. planes.
Refinement. Refinement of *F*^2^ against ALL reflections. The weighted *R*-factor *wR* and goodness of fit *S* are based on *F*^2^, conventional *R*-factors *R* are based on *F*, with *F* set to zero for negative *F*^2^. The threshold expression of *F*^2^ > σ(*F*^2^) is used only for calculating *R*-factors(gt) *etc*. and is not relevant to the choice of reflections for refinement. *R*-factors based on *F*^2^ are statistically about twice as large as those based on *F*, and *R*- factors based on ALL data will be even larger.Several crystal specimens were tested but their quality was, in general, rather poor, as witnessed by the relatively high fraction of low intensity reflections. The poorly diffracting nature of the crystals is the reason for the relatively high *R* factors.

### Fractional atomic coordinates and isotropic or equivalent isotropic displacement parameters (Å^2^)

**Table:**

	*x*	*y*	*z*	*U*_iso_*/*U*_eq_	
O1	0.5087 (7)	0.2972 (5)	0.6271 (4)	0.0695 (14)	
O2	0.7415 (7)	0.2144 (5)	0.5641 (3)	0.0546 (13)	
H2	0.828 (10)	0.252 (7)	0.625 (5)	0.065*	
O3	−0.6820 (6)	−0.1393 (4)	0.2211 (3)	0.0449 (11)	
H3	−0.791 (9)	−0.207 (6)	0.168 (4)	0.054*	
O4	−0.4973 (6)	−0.2424 (4)	0.1314 (3)	0.0579 (13)	
N1	0.0479 (6)	0.1074 (4)	0.3096 (3)	0.0347 (11)	
N2	0.1576 (6)	0.3969 (4)	0.0245 (3)	0.0330 (11)	
N3	0.0616 (7)	0.3422 (4)	−0.0794 (4)	0.0351 (11)	
N4	0.2872 (7)	0.5635 (5)	−0.0763 (4)	0.0321 (12)	
H4	0.327 (8)	0.603 (6)	−0.010 (4)	0.039*	
N5	0.0928 (6)	0.3985 (4)	−0.2380 (3)	0.0350 (11)	
N6	0.2377 (9)	0.5392 (6)	−0.4342 (5)	0.0736 (18)	
N7	0.5606 (7)	0.8734 (5)	−0.1101 (4)	0.0530 (15)	
C1	0.5542 (10)	0.2307 (6)	0.5524 (5)	0.0443 (16)	
C2	0.4013 (8)	0.1581 (6)	0.4388 (4)	0.0410 (15)	
H2A	0.3851	0.0586	0.4165	0.049*	
H2B	0.4604	0.1965	0.3898	0.049*	
C3	0.1838 (8)	0.1715 (6)	0.4252 (4)	0.0390 (14)	
H3A	0.1175	0.1254	0.4685	0.047*	
H3B	0.1990	0.2707	0.4522	0.047*	
C4	−0.5053 (8)	−0.1506 (6)	0.2034 (4)	0.0360 (14)	
C5	−0.3145 (8)	−0.0271 (6)	0.2831 (4)	0.0443 (15)	
H5A	−0.3144	−0.0190	0.3570	0.053*	
H5B	−0.3294	0.0582	0.2714	0.053*	
C6	−0.1030 (8)	−0.0340 (6)	0.2757 (4)	0.0379 (14)	
H6A	−0.0501	−0.0828	0.3226	0.045*	
H6B	−0.1197	−0.0867	0.2007	0.045*	
C7	0.0604 (7)	0.1772 (6)	0.2383 (4)	0.0327 (13)	
C8	−0.0607 (8)	0.1135 (5)	0.1257 (4)	0.0327 (13)	
H8	−0.1607	0.0232	0.1005	0.039*	
C9	−0.0354 (7)	0.1804 (5)	0.0534 (4)	0.0318 (13)	
H9	−0.1138	0.1331	−0.0208	0.038*	
C10	0.1061 (7)	0.3192 (5)	0.0874 (4)	0.0319 (13)	
C11	0.2172 (8)	0.3865 (5)	0.2009 (4)	0.0354 (14)	
H11	0.3079	0.4797	0.2262	0.042*	
C12	0.1978 (8)	0.3213 (5)	0.2750 (4)	0.0350 (13)	
H12	0.2732	0.3700	0.3494	0.042*	
C13	0.1445 (7)	0.4320 (5)	−0.1300 (4)	0.0303 (13)	
C14	0.3292 (8)	0.6199 (5)	−0.1517 (4)	0.0342 (14)	
C15	0.2101 (8)	0.5140 (6)	−0.2531 (4)	0.0371 (14)	
C16	0.4603 (8)	0.7600 (6)	−0.1284 (4)	0.0382 (14)	
C17	0.2170 (9)	0.5250 (6)	−0.3564 (5)	0.0457 (16)	

### Atomic displacement parameters (Å^2^)

**Table:**

	*U*^11^	*U*^22^	*U*^33^	*U*^12^	*U*^13^	*U*^23^
O1	0.074 (3)	0.066 (3)	0.041 (3)	0.025 (3)	−0.002 (2)	−0.005 (2)
O2	0.041 (3)	0.058 (3)	0.042 (3)	0.008 (2)	−0.007 (2)	0.010 (2)
O3	0.031 (2)	0.048 (3)	0.039 (2)	−0.0005 (19)	0.0067 (19)	0.007 (2)
O4	0.051 (3)	0.051 (3)	0.040 (2)	0.001 (2)	0.010 (2)	−0.013 (2)
N1	0.031 (2)	0.030 (3)	0.029 (3)	−0.002 (2)	0.001 (2)	0.008 (2)
N2	0.030 (2)	0.030 (3)	0.030 (3)	0.005 (2)	0.007 (2)	0.005 (2)
N3	0.037 (3)	0.030 (3)	0.034 (3)	0.007 (2)	0.012 (2)	0.009 (2)
N4	0.029 (2)	0.025 (3)	0.030 (2)	0.001 (2)	0.006 (2)	0.003 (2)
N5	0.027 (2)	0.030 (3)	0.035 (3)	0.003 (2)	−0.001 (2)	0.010 (2)
N6	0.074 (4)	0.083 (5)	0.053 (4)	0.009 (3)	0.015 (3)	0.029 (3)
N7	0.044 (3)	0.040 (3)	0.067 (4)	0.005 (3)	0.020 (3)	0.015 (3)
C1	0.052 (4)	0.030 (4)	0.037 (4)	0.005 (3)	0.002 (3)	0.010 (3)
C2	0.041 (3)	0.040 (4)	0.033 (3)	0.007 (3)	0.003 (3)	0.012 (3)
C3	0.033 (3)	0.041 (4)	0.033 (3)	0.001 (3)	0.007 (3)	0.011 (3)
C4	0.034 (3)	0.038 (4)	0.031 (3)	0.004 (3)	0.007 (3)	0.015 (3)
C5	0.039 (3)	0.040 (4)	0.039 (3)	0.000 (3)	0.011 (3)	0.005 (3)
C6	0.034 (3)	0.035 (3)	0.034 (3)	0.002 (3)	0.005 (3)	0.010 (3)
C7	0.021 (3)	0.029 (3)	0.035 (3)	0.005 (2)	0.005 (2)	0.000 (3)
C8	0.027 (3)	0.026 (3)	0.036 (3)	0.002 (2)	0.006 (2)	0.007 (3)
C9	0.029 (3)	0.024 (3)	0.030 (3)	−0.002 (2)	0.002 (2)	0.007 (2)
C10	0.022 (3)	0.032 (3)	0.032 (3)	0.009 (2)	0.002 (2)	0.002 (3)
C11	0.033 (3)	0.025 (3)	0.040 (3)	0.002 (2)	0.012 (3)	0.006 (3)
C12	0.035 (3)	0.033 (3)	0.024 (3)	0.006 (3)	0.005 (2)	0.000 (2)
C13	0.024 (3)	0.033 (3)	0.028 (3)	0.008 (2)	0.006 (2)	0.006 (2)
C14	0.029 (3)	0.031 (3)	0.036 (3)	0.005 (3)	0.008 (2)	0.008 (3)
C15	0.028 (3)	0.042 (4)	0.031 (3)	0.007 (3)	0.003 (2)	0.008 (3)
C16	0.031 (3)	0.038 (4)	0.045 (4)	0.011 (3)	0.013 (3)	0.014 (3)
C17	0.040 (3)	0.049 (4)	0.037 (4)	0.004 (3)	0.006 (3)	0.014 (3)

### Geometric parameters (Å, º)

**Table:**

O1—C1	1.213 (7)	C2—H2B	0.9700
O2—C1	1.323 (7)	C3—H3A	0.9700
O2—H2	0.80 (6)	C3—H3B	0.9700
O3—C4	1.324 (7)	C4—C5	1.502 (7)
O3—H3	0.88 (5)	C5—C6	1.504 (7)
O4—C4	1.187 (6)	C5—H5A	0.9700
N1—C7	1.350 (7)	C5—H5B	0.9700
N1—C6	1.449 (6)	C6—H6A	0.9700
N1—C3	1.465 (6)	C6—H6B	0.9700
N2—N3	1.278 (5)	C7—C8	1.408 (7)
N2—C10	1.361 (6)	C7—C12	1.434 (7)
N3—C13	1.384 (6)	C8—C9	1.353 (7)
N4—C13	1.346 (6)	C8—H8	0.9300
N4—C14	1.347 (7)	C9—C10	1.404 (7)
N4—H4	0.81 (5)	C9—H9	0.9300
N5—C13	1.325 (6)	C10—C11	1.408 (7)
N5—C15	1.363 (6)	C11—C12	1.356 (7)
N6—C17	1.127 (7)	C11—H11	0.9300
N7—C16	1.133 (6)	C12—H12	0.9300
C1—C2	1.483 (7)	C14—C15	1.392 (7)
C2—C3	1.514 (7)	C14—C16	1.415 (8)
C2—H2A	0.9700	C15—C17	1.427 (8)
			
C1—O2—H2	116 (5)	N1—C6—C5	110.1 (4)
C4—O3—H3	108 (4)	N1—C6—H6A	109.6
C7—N1—C6	121.5 (4)	C5—C6—H6A	109.6
C7—N1—C3	121.7 (4)	N1—C6—H6B	109.6
C6—N1—C3	116.7 (4)	C5—C6—H6B	109.6
N3—N2—C10	118.0 (4)	H6A—C6—H6B	108.2
N2—N3—C13	109.7 (4)	N1—C7—C8	122.1 (5)
C13—N4—C14	108.1 (4)	N1—C7—C12	120.9 (5)
C13—N4—H4	124 (4)	C8—C7—C12	117.0 (5)
C14—N4—H4	127 (4)	C9—C8—C7	121.7 (5)
C13—N5—C15	105.1 (4)	C9—C8—H8	119.1
O1—C1—O2	123.8 (6)	C7—C8—H8	119.1
O1—C1—C2	122.5 (6)	C8—C9—C10	121.7 (5)
O2—C1—C2	113.7 (6)	C8—C9—H9	119.1
C1—C2—C3	114.2 (5)	C10—C9—H9	119.1
C1—C2—H2A	108.7	N2—C10—C9	128.5 (5)
C3—C2—H2A	108.7	N2—C10—C11	114.8 (4)
C1—C2—H2B	108.7	C9—C10—C11	116.6 (5)
C3—C2—H2B	108.7	C12—C11—C10	122.9 (5)
H2A—C2—H2B	107.6	C12—C11—H11	118.6
N1—C3—C2	111.0 (4)	C10—C11—H11	118.6
N1—C3—H3A	109.4	C11—C12—C7	119.8 (5)
C2—C3—H3A	109.4	C11—C12—H12	120.1
N1—C3—H3B	109.4	C7—C12—H12	120.1
C2—C3—H3B	109.4	N5—C13—N4	111.6 (5)
H3A—C3—H3B	108.0	N5—C13—N3	123.9 (4)
O4—C4—O3	125.1 (5)	N4—C13—N3	124.5 (4)
O4—C4—C5	123.8 (5)	N4—C14—C15	105.3 (5)
O3—C4—C5	111.0 (5)	N4—C14—C16	125.5 (5)
C4—C5—C6	115.5 (5)	C15—C14—C16	129.1 (5)
C4—C5—H5A	108.4	N5—C15—C14	109.8 (5)
C6—C5—H5A	108.4	N5—C15—C17	125.8 (5)
C4—C5—H5B	108.4	C14—C15—C17	124.4 (5)
C6—C5—H5B	108.4	N7—C16—C14	178.1 (6)
H5A—C5—H5B	107.5	N6—C17—C15	175.0 (6)
			
C10—N2—N3—C13	−175.9 (4)	C9—C10—C11—C12	2.4 (8)
O1—C1—C2—C3	−1.3 (8)	C10—C11—C12—C7	0.5 (8)
O2—C1—C2—C3	177.9 (5)	N1—C7—C12—C11	176.1 (5)
C7—N1—C3—C2	−81.8 (6)	C8—C7—C12—C11	−4.6 (7)
C6—N1—C3—C2	99.1 (5)	C15—N5—C13—N4	0.6 (6)
C1—C2—C3—N1	175.3 (5)	C15—N5—C13—N3	−179.4 (5)
O4—C4—C5—C6	−7.2 (8)	C14—N4—C13—N5	0.9 (6)
O3—C4—C5—C6	175.8 (5)	C14—N4—C13—N3	−179.2 (5)
C7—N1—C6—C5	−85.4 (6)	N2—N3—C13—N5	172.9 (5)
C3—N1—C6—C5	93.7 (6)	N2—N3—C13—N4	−7.0 (7)
C4—C5—C6—N1	150.8 (5)	C13—N4—C14—C15	−2.0 (6)
C6—N1—C7—C8	−4.7 (8)	C13—N4—C14—C16	175.5 (5)
C3—N1—C7—C8	176.2 (5)	C13—N5—C15—C14	−1.8 (6)
C6—N1—C7—C12	174.7 (5)	C13—N5—C15—C17	176.3 (6)
C3—N1—C7—C12	−4.4 (7)	N4—C14—C15—N5	2.4 (6)
N1—C7—C8—C9	−174.8 (5)	C16—C14—C15—N5	−175.0 (5)
C12—C7—C8—C9	5.8 (8)	N4—C14—C15—C17	−175.8 (5)
C7—C8—C9—C10	−2.9 (8)	C16—C14—C15—C17	6.9 (10)
N3—N2—C10—C9	3.2 (8)	N4—C14—C16—N7	−92 (19)
N3—N2—C10—C11	−179.6 (5)	C15—C14—C16—N7	85 (19)
C8—C9—C10—N2	175.9 (5)	N5—C15—C17—N6	−143 (8)
C8—C9—C10—C11	−1.3 (7)	C14—C15—C17—N6	35 (9)
N2—C10—C11—C12	−175.1 (5)		

### Hydrogen-bond geometry (Å, º)

**Table:**

*D*—H···*A*	*D*—H	H···*A*	*D*···*A*	*D*—H···*A*
O2—H2···N5^i^	0.80 (6)	2.12 (6)	2.896 (6)	162 (6)
O3—H3···N3^ii^	0.88 (5)	1.89 (6)	2.750 (6)	165 (5)
N4—H4···O4^iii^	0.81 (5)	1.98 (5)	2.740 (6)	156 (5)

Symmetry codes: (i) *x*+1, *y*, *z*+1; (ii) −*x*−1, −*y*, −*z*; (iii) *x*+1, *y*+1, *z*.
